# Deep features optimization based on a transfer learning, genetic algorithm, and extreme learning machine for robust content-based image retrieval

**DOI:** 10.1371/journal.pone.0274764

**Published:** 2022-10-03

**Authors:** Ruqia Bibi, Zahid Mehmood, Asmaa Munshi, Rehan Mehmood Yousaf, Syed Sohail Ahmed

**Affiliations:** 1 University Institute of Information Technology, Pir Mehr Ali Shah Arid Agriculture University, Rawalpindi, Pakistan; 2 Department of Computer Engineering, University of Engineering and Technology, Taxila, Pakistan; 3 College of Computer Science and Engineering, University of Jeddah, Jeddah, Saudi Arabia; 4 Department of Computer Engineering, College of Computer, Qassim University, Buraydah, Saudi Arabia; IIT Madras, INDIA

## Abstract

The recent era has witnessed exponential growth in the production of multimedia data which initiates exploration and expansion of certain domains that will have an overwhelming impact on human society in near future. One of the domains explored in this article is content-based image retrieval (CBIR), in which images are mostly encoded using hand-crafted approaches that employ different descriptors and their fusions. Although utilization of these approaches has yielded outstanding results, their performance in terms of a semantic gap, computational cost, and appropriate fusion based on problem domain is still debatable. In this article, a novel CBIR method is proposed which is based on the transfer learning-based visual geometry group (VGG-19) method, genetic algorithm (GA), and extreme learning machine (ELM) classifier. In the proposed method, instead of using hand-crafted features extraction approaches, features are extracted automatically using a transfer learning-based VGG-19 model to consider both local and global information of an image for robust image retrieval. As deep features are of high dimension, the proposed method reduces the computational expense by passing the extracted features through GA which returns a reduced set of optimal features. For image classification, an extreme learning machine classifier is incorporated which is much simpler in terms of parameter tuning and learning time as compared to other traditional classifiers. The performance of the proposed method is evaluated on five datasets which highlight the better performance in terms of evaluation metrics as compared with the state-of-the-art image retrieval methods. Its statistical analysis through a nonparametric Wilcoxon matched-pairs signed-rank test also exhibits significant performance.

## 1. Introduction

**Fig 1:**
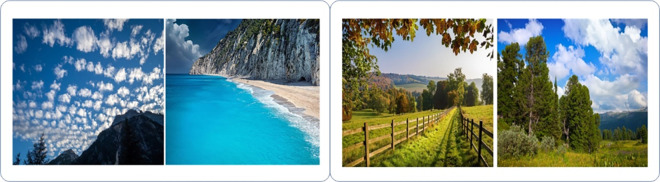
Images having similar visual appearance but belongs to different classes [3] (reprinted from [3] under a CC BY license, with permission from J. Z. WANG, original copyright [2003]).

People nowadays love to capture and share their life happenings e.g. via social media platforms which leads to the extensive growth of multimedia data, it triggers the need for certain techniques that can allow people to store, filter, or retrieve data whenever a need arises [1]. In the case of images, these techniques must provide an image representation that can be used to effectively classify images according to their similar visual representations. A content-based image retrieval system (CBIR) uses the content of an image to retrieve images from datasets having similar visual representations. Here visual representation depicts the color, texture, or shape of an image. A typical CBIR system works by transforming training images into corresponding feature vectors through techniques that can be both hand-crafted and based on deep learning approaches [2]. A query image is then fed to the system where its feature vector is compared against the feature dataset and similar images are retrieved based on the similarity scores. The appropriate selection of image representation methods, classifiers, and similarity measures is crucial for the success of image retrieval systems. In literature, researchers have explored and presented many new techniques based on handcrafted features or deep features to classify images, but it remains a challenging task because of the semantic gap (Fig 1), a variance that exists between low-level image representations and human level semantics.

Handcrafted feature extraction approaches use descriptors to detect and describe objects of interest within images. Several studies combine these descriptors to enhance the retrieval accuracy of the CBIR system. These strategies have performed remarkably well in expressing the image contents but still, their image expressing capabilities are limited, expensive to design, and hard to transfer learned knowledge to new domains on larger size image datasets. An alternative to handcrafted features is machine learning approaches that can learn features on their own and have better description capabilities. Studies in the recent past [4, 5] have reported remarkable results of deep learning in terms of accuracy, precision, and applicability in diverse areas. The convolutional neural networks (CNN) based methods not only enhance the classification accuracy but are now assessed to be good generic feature descriptors. CNN extract features hierarchically where lower layers encode lower features i.e. edges, shapes, texture, etc. while higher layers encode semantic level details of an image. These networks do not require any pre-processing as kernels are learned rather than handcrafted hence no initial parameterization and human intervention are needed. Its huge success has overwhelmed the researchers but its reliance on a huge amount of data, low feature interpretability [2], longer training time, and requirement of massive computational resources are some of the prominent limitations. To address the above limitations, the proposed method uses the VGG-19 model to extract features that have both global and local information contained by an image. Features from the FC 7 layer of the VGG-19 model are utilized. To further optimize the extracted features and reduce the computational expense, a genetic algorithm is employed. Afterward, images are classified through an extreme learning machine classifier which is a single layer feed-forward neural network having a shorter learning time and robust in terms of convergence and generalization as compared to alternate classification methods like support vector machine (SVM), Boltzmann machine (BM), restricted Boltzmann machine (RBM), deep belief network (DBN), Hopfield neural network (HNN), etc. [6, 7].

The main contributions of the proposed method are as follows:

- An optimized feature set is constructed by applying a genetic algorithm over-extracted deep features from VGG-19 architecture for robust CBIR and to reduce the computational expense of the proposed method.
- The semantic gap issue of CBIR is reduced between the extracted features and high-level semantic concepts of the images.
- For efficient, effective learning and convergence, an ELM classifier is utilized for the proposed method.
- An extensive experimental analysis over five datasets (namely Wang-A, Wang-B, OT Scene, Wang 10k, and Caltech 256) is conducted to examine the scalability of the proposed method as compared with state-of-the-art CBIR methods.

The rest of the paper is organized as follows: Section 2 highlights some of the existing related work. Section 3 presents the proposed methodology in detail. Experimental discussion and achieved results are provided in section 4. Section 5 presents the conclusion and future direction of the research.

## 2. Related works

Fadaei et al. [8] address issues such as noise and image translation by integrating several wavelets and curvelet features along with the dominant color descriptor (DCD). Firstly, HSV color space is considered because of its ability to differentiate chromatic and achromatic components precisely. The extraction of DCD features from HSV color space resulted in coarse partitions. So, to get even partitions, pixels are classified based on similar probability. After that, corresponding centers are defined based on their distances, not their partitions, to yield better accuracy. Meanwhile, the combination of the Frobenius norm with wavelet and curvelet transform is proposed for texture representation. Grouping of three feature sets using a particle swarm optimization algorithm showed better performance than competitor methods even with more running time. Images should not only be compared depending on their regions but also on their nature because if the regions are considered only, the accuracy of the system would be inefficient. Considering this, image retrieval based on location-independent ROI is presented by Raghuwanshi et al. [9]. This novel approach segments an image into the texture and non-textured region. Tetrolet transform is used to highlight texture regions and for non-textured regions moment invariants in combination with the edge, features are used. Varying block sizes are used for finding optimal blocks for segmentation. A larger block size resulted in overlapping regions and increased segmentation time, therefore an 8×8 block is suggested. A similarity count is added to give a higher rank to images having more similar regions hence reducing no. of comparisons and better precision and recall. Another image representation method based on iterative DCT and sparse representation is presented in [10]. HSI and CIE-LAB color spaces are analyzed because of their uniform color perception which is considered to be related to human perception. Sparse representation is combined with several available acceleration techniques like DALM, PALM, etc. to investigate retrieval results. The proposed method’s performance is evaluated by varying recall probability and averaged modified normalized retrieval rank. Experimental analysis shows a remarkable reduction in storage requirements and vector size.

To identify prominent objects with high precision in an image Rehan et al. [11] proposed a novel image representation method based on color histogram and bandelets transform. The proposed method highlights the most edifying texture regions and uses artificial neural networks to overcome incorrect geometric classification. After determining the semantic class of an image by SVM, a reverted index mechanism used by google for text-based search is also incorporated for fast image retrieval. Experimental evaluation shows promising results without external management from a user as with many relevant feedbacks based CBIR systems. If a machine vision system can identify salient objects in an image in the early stages of recognition, it will be possible to not only generate proper object detection windows for further processing but will also reduce computational costs to much extent. Considering this a method for salient object subitizing (SOS) combined with CNN is presented by Zhang et al. [12]. For the training of CNN based SOS model, 20k synthetic images are generated by varying no. of salient objects and background images. This method successfully suppresses false object detection and results in better average precision for images having 3 dominant objects. Hussain et al. [13] present an improved pre-processing technique using Quaternion transform to highlight salient regions of the image thereby improving the retrieval rate.

Anandh et al. [36] presented a hybrid framework comprising local features for CBIR. The framework uses color auto-correlogram, Gabor wavelet, and wavelet transform for extracting color, texture, and shape simultaneously. For deriving texture, six orientations and four scales are used. This method used SVM as a classifier and Manhattan distance as a similarity measure for image retrieval. In terms of performance, the combination of hybrid features resulted in improved performance as compared to the individual feature representation methods for image retrieval. Dubey et al. [37] have come up with a novel descriptor based on adder and decoder concepts. Local binary patterns (LBP) of three channels are combined with adder and decoder to yield outputs of 3 input channels, 4 adder channels, and 8 decoder channels. In terms of performance, decoder channels highlight color texture information better as compared to adders and input channels. A higher dimension of the feature vector is one of the shortcomings of decoder-based LBP. A robust feature representation model based on local texton XOR patterns (LTxXORP) is presented by Bala et al [38]. The proposed model divides the V space of HSV color space into sub-blocks of 2×2. Texton images are generated by applying 7 texton shapes on each sub-block. Afterward, an XOR operation is performed between the center pixels and neighboring pixels of the resultant image. Histograms of HSV color spaces and the texton XOR image are concatenated to get the final feature vector. The experimental analysis highlights the robustness of the proposed model as compared to other LBP-based methods. Another novel method based on bag-of-words (BoW) is presented by Sarwar et al. [39] to address the semantic gap issue that occurred in a CBIR system. The proposed method builds a dictionary that incorporates complementary features from both LBPV and LIOP descriptors by applying density-based spatial clustering of applications with the noise (DBSCAN) method. LBPV features overcame the loss of global texture information faced by LBP by adding variance as a weight to get the feature vector. On the contrary, to preserve both local and global order of pixel intensities, LIOP is used. The dimensions of a resulted feature vector are reduced using PCA and classification is performed using SVM. The experimental analysis highlights better recall by forming a small-size visual dictionary and better precision by forming a large-size dictionary. `In most of the studies to represent features, the output of the last layers of a single CNN without quantization is used. Hence, the intermediate convolution layers remain neglected. To address this Alzu’bi et al. [40] have proposed a bilinear approach named CRB-CNN by modeling two CNNs in parallel i.e. VGG-16 and VGG-m for extracting features from intermediate convolution layers in an unsupervised way, which resulted in low dimension but compact and highly discriminative features of vector length 16. This method uses the first 15 and 30 layers of VGG-m and VGG-16 respectively and replaced fully connected layers with three new layers i.e. root pooling, sqrt, and L2 normalization. The model reduces the dimensions of image features into several compact dimensions i.e. 512,128, 64,32,16. The experimental analysis highlights the best retrieval accuracy overall dimensions when Euclidean distance is used as a similarity measure. In the case of Manhattan distance, accuracy tends to improve when vector size is set to 64 and starts to degrade when size is reduced to 32 or 16. In [41] Quantization as the pre-processing step is also suggested to reduce dimensions.

Mary et al. [42] presented a hybrid feature selection method based on a genetic algorithm. The feature set is a merger of color moments, entropy, energy, homogeneity, contrast, and feature descriptor. A backpropagation neural network is used as a feature selection algorithm as well as for classification. 10 best features are selected from a set of 26 features. The approach’s performance is judged considering many similarity measures but the modified normalized retrieval rank evaluated the system accurately. Using CNN for feature extraction is also suggested by Shah et al. [43] based on the precision achieved against competitive methods. Bai et al. [44] have come up with an optimized version of AlexNet named (OANIR). The proposed improvements of this method are a combination of max and average pooling, the use of maxout function as the activation function for fully connected layers, and the addition of a hidden layer for binary code representation. At a hidden layer, a binary code function is used to limit output between 0 and 1. Extracted and queried binary codes are then judged based on hamming distance. OANIR has outperformed the original AlexNet in terms of precision and mAP even for large-scale image datasets. Can the same binary code be used for retrieval and compression to efficiently utilize storage? To answer this Zhang et al. [45] have studied deep networks for image compression. Two deep networks are trained. First, for representation of the image in compressed bitstream form, and second for extracting features. Both the trained networks are then combined using triplets of images. The proposed method outperformed in terms of JPEG compression and achieved a compression ratio of 5.3 for 32×32 thumbnails. The performance of several classifiers i.e. SVM, LSSVM, NN, ELM, and kernel ELM for the object recognition domain is evaluated by Zhang et al. [46]. The deep features are extracted using CNN having 5 convolutions and 3 fully connected layers pre-trained on the ImageNet dataset. Layers 6 and 7 are used as inputs for classification. The recognition accuracies are tested under three setups i.e. single domain, cross-domain using source, and cross-domain using source and target. In all three setups, kernelized ELM shows a state-of-the-art performance among all. Recent advances in CBIR are comprehended in [47]. The study highlights key challenges in generic modules of the CBIR framework and suggested a variety of representative strategies and methods to overcome recognized challenges. Guo et al. [48] describe various deep learning approaches comprehensively and summarizes the significant issues related to the design and training of deep networks. The study provides insight into the scope and compares the performance of deep networks on commonly used datasets. The details of competitive CBIR methods are presented in Table 1.

**Table 1:** Details of competitive CBIR methods.

Technique	Problem addressed	Feature extraction	Clustering	Classification	Similarity measure	Limitations/ Future work
**FIF-IRS [14]**	Semantic gap, the computational cost	8-Directional Gray Level Co-occurrence Matrix, geometric shape features, and HSV Color Moments	N.A.	N.A.	Manhattan, Canberra Euclidean and statistical distance	To integrate optimization techniques to reduce dimensions of feature vectors
**SCNN-ELM [15]**	Classification accuracy	Fine-tune AlexNet	N.A.	Extreme learning machine	N.A.	Misclassified visually similar images
**CM-LBP-CED [16]**	Retrieval accuracy	Color moments, Local Binary Pattern (LBP) and Canny edge detection	N.A.	N.A.	Manhattan distance	Incorporate deep learning techniques
**GMM-mSpatiogram [17]**	Lack of spatial information, dimensionality problem	GMM based color quantization method, spatiograms	Expectation maximization-Bayesian Information Criterion	N.A.	Mahalanobis distance, Jensen–Shannon Divergence	To explore more sophisticated color and texture information
**SIFT-SURF [18]**	Semantic gap	SIFT, SURF	k-means	SVM	Euclidean distance	Incorporate deep learning techniques
**CM-DWT-CEDD [19]**	Semantic gap	Color moments, Gabor and Discrete wavelet transform, Color and Edge Directivity Descriptor	N.A.	N.A.	Euclidean distance	Incorporate deep learning-based classifiers
**PUD [20]**	Incompatibility of image descriptor and ranking methods	Perceptual Uniform Descriptor	N.A.	N.A.	L1/L2 norm, Scatter balance metric learning	Manifold ranking with multi-graph fusion
**N3G-MFR [21]**	Role of image re-ranking in CBIR	HSV, SIFT, AlexNet	N.A.	N.A.	Jaccard similarity	To incorporate feature extraction and fusion re-ranking
**ResNet [22]**	Vanishing gradient	Residual network	N.A.	Minimum distance classifier	Canberra distance	Use of deep architecture in the medical field
**CDH-ART [23]**	Fusion framework for ranking retrieval results	Color Difference Histogram and Angular Radial Transform features	N.A.	N.A.	Euclidean distance, Modified Canberra distance	Handcrafted features, computationally expensive
**B-T-Morph [24]**	Semantic gap	Image binarization, image transform, and morphological operator	N.A.	ANN, SVM	Euclidean distance, City block distance	Handcrafted features, computationally expensive
**HWVP [25]**	Semantic gap, Effective feature representation	Hierarchical wavelet packet descriptors	N.A.	SVM	Euclidean distance	Over partitioning of images leads to disrupted texture patterns
**ISA-SPM [26]**	Learning difficulty in dynamic image samples	Independent Subspace Analysis-spatial pyramid matching	k-means	SVM	Histogram intersection	Sensitive to noise, required fixed group size for random vectors
**FC-GPHOG [27]**	Challenges in object and scene image classification	GP-HOG, FC-GPHOG, enhanced fisher model	N.A.	Nearest neighbor	Cosine similarity	To handle fuzzy memberships of class images.
**Balanced tree structures [28]**	Computational complexities in case of a large no. of classes	Dense SIFT, Locally constrained linear coding, Spatial pyramid matching	k-means	One vs all binary classifiers	N.A.	Incorporation of the semantic relationship between classes and distribution of classes
**ResNet-HAM [29]**	Lack of prior knowledge while transferring to a new domain .	ResNet-50, VGG-16	k-means	Hopfield network	Euclidean distance between two weighted matrices	Inherent ambiguity while retrieving images from certain classes.
**ILHS [30]**	Visual similarity vs semantic correlation	NA	Spectral clustering	SVM	Euclidean distance	Incorporate CNN-based representations for better classification accuracies.
**Spatial color-Shape [31]**	Lack of spatial information, dimensionality reduction	BRISK like FREAK, Spatial CH, BoW	N.A.	K-nearest neighbors	Chebyshev distance	To incorporate scale invariancy
**SURF-HOG [32]**	Semantic gap	SURF, HOG	k-means++	SVM	Euclidean distance	To incorporate spatial information
**CHLDP-DSIFT [33]**	Image diffusion	CH, LDP, SIFT, BoF	N.A.	K- nearest neighbors	Manhattan distance	Time optimization of a diffusion process
**MDGHM-SURF-ORB [34]**	Semantic gap	MDGHM-SURF-ORB	Fuzzy c- means	Soft label SVM	Canberra distance	Incorporate VLAD, deep learning approaches
**DNN-SAR [35]**	Semantic gap	Local binary pattern, Zernike moments, HSV histogram	Adaptive Sunflower optimization algorithm (SFO)	Deep neural network-search and rescue optimization algorithm (DNN- SAR)	Matching difference	Integration of Hadoop approaches with CBIR
**Proposed method**	Semantic gap, dimensionality reduction, robust feature representation, the computational expense	Transfer learning based on VGG-19 architecture, GA	N.A.	ELM	Canberra distance	Incorporate other deep learning techniques

## 3. The methodology of the proposed model

**Fig 2:**
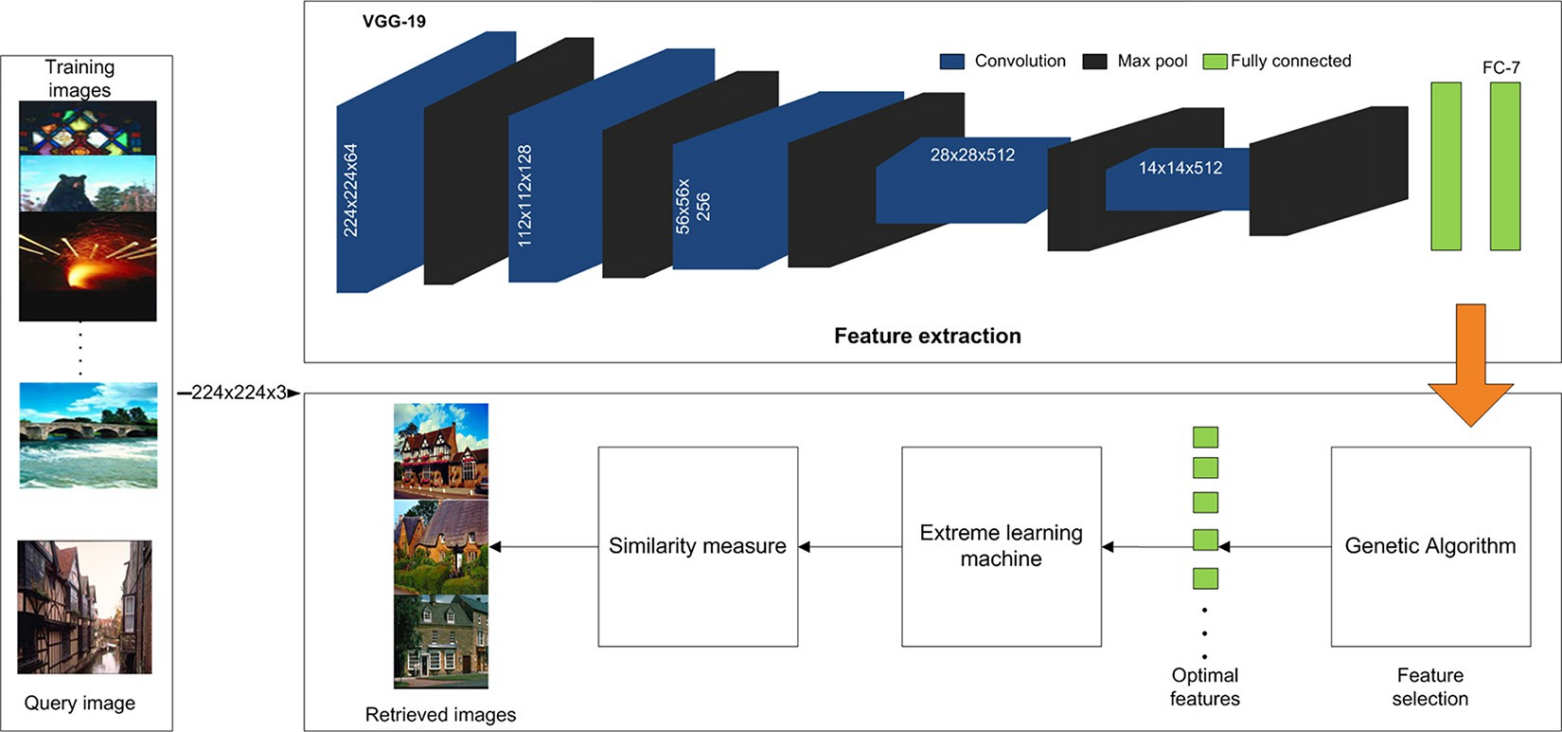
A proposed methodology for CBIR (reprinted from [3] under a CC BY license, with permission from J. Z. WANG, original copyright [2003]).

This section discusses in detail the methodology of the proposed method as presented in Fig 2. The three primary steps of the proposed methodology are a) deep feature learning through transfer learning and VGG-19, b) selection of optimal features and c) image classification using ELM. A similarity between a query image and training images are judged based on Canberra distance. A detailed description of each of these steps is presented in subsequent sections.

### 3.1 Features learning

Machine learning algorithms have always worked by mapping the relationship between input and output data based on the learned knowledge. In case the input/training data shares the same feature space or distribution as output/testing data, the predictions would be accurate. On the contrary, if they belong to different feature spaces then the predicted outcomes would be inaccurate hence, degrades the overall performance of the system. As mentioned earlier, with the exponential growth of image repositories, utilizing an existing dataset not entirely similar but close to the target domain seems an efficient approach. Hence, in the proposed method transfer learning (TL) is being employed for optimizing time and resources by fine-tuning or utilizing a pre-trained network. TL is defined in [49] as given a source domain *S* and learning Task *L*_*S*_, a target domain *T* and learning task *L*_*T*_, transfer learning improves the learning of the target predictive function *f*_*T*_(∙) in *T* by utilizing the knowledge in *S* and *L*_*S*_, where *S*≠*T* and *L*_*s*_≠*L*_*T*_. When utilizing a pre-trained network, parameters of the initial layers are used as it is rather than initializing the parameters randomly which enhances the generalization ability of the model and accelerates the learning process.

In the proposed method, VGG-19 architecture (discussed in subsequent sections) is retrained on our selected datasets. As this network intake images of a specific size so, preprocessing of the images is being done to make them compatible with the network’s initial layers. In VGG-19, after training the network, the fully connected layer 7 is considered as a feature map having a 4096×1 dimensional vector.

#### 3.1.1 CNN architecture for the proposed method

A convolution neural network (CNN) starts with *N* number of training images, which are passed through several convolution layers followed by some pooling layers to the final fully connected layers. In convolution layers, features are extracted by convolving filters *f* of size *m*×*m* with image *I* at all spatial locations. A linear convolution operation outputs a feature map having distinct details and is smaller in size than the original image. Mathematically, a feature value in *f*^*th*^ feature map of layer *l* at location (*x*, *y*) is expressed as:

$$F_{x,y,f}^{l}={w_{f}^{l}}^{T}x_{x,y}^{l}+b_{f}^{l}$$ (1)

where *x*, *b*, and *w* represent input patch, bias, and weight vector, respectively. To detect non-linear features, activation functions like ReLu [50], sigmoid, and tanh are mostly used to add non-linearity to CNN. ReLU activation function is expressed as:

ReLU=max(0,x) (2)

Early layers of CNN capture local details i.e. edges, curves, textures, etc. while as the layer gets deeper and deeper these networks can have a semantic level understanding of images like we humans do. Upper layers of CNN are also referred to by [4] as good descriptors. The number of kernels, stride factor and size are some of the parameters of convolution layers.

Stacked feature maps are then passed to pooling layers that reside between succeeding convolution layers to reduce the overall computation burden through a reduction in no. of trainable parameters. In other words, pooling layers reduce the dimension of feature maps by applying a downsample operation hence, achieving translation invariance. Max, min, and average pooling are some variant operations of this layer. For a pooling region of size *n*×*n*, max-pooling can be mathematically expressed as:

$${MX}_{j}^{l}=\frac{max}{1\leq i\leq n\times n}(x_{j})$$ (3)

where ${MX}_{j}^{l}$ represents the output of max-pooling operation at layer *l* using *n*×*n* pooling region. After passing through a series of multiple convolutions and pooling layers, the resultant output is then flattened into a single-dimensional vector which determines the probability of possible class labels. All the neurons of the previous layer are connected to each neuron of a fully connected layer to predict semantic association to a class. A loss function is then used to measure the prediction error. Once an error is calculated, results are backpropagated to update weights and biases to reduce misclassification.

#### 3.1.2 VGG -19 for the proposed method

**Fig 3:**
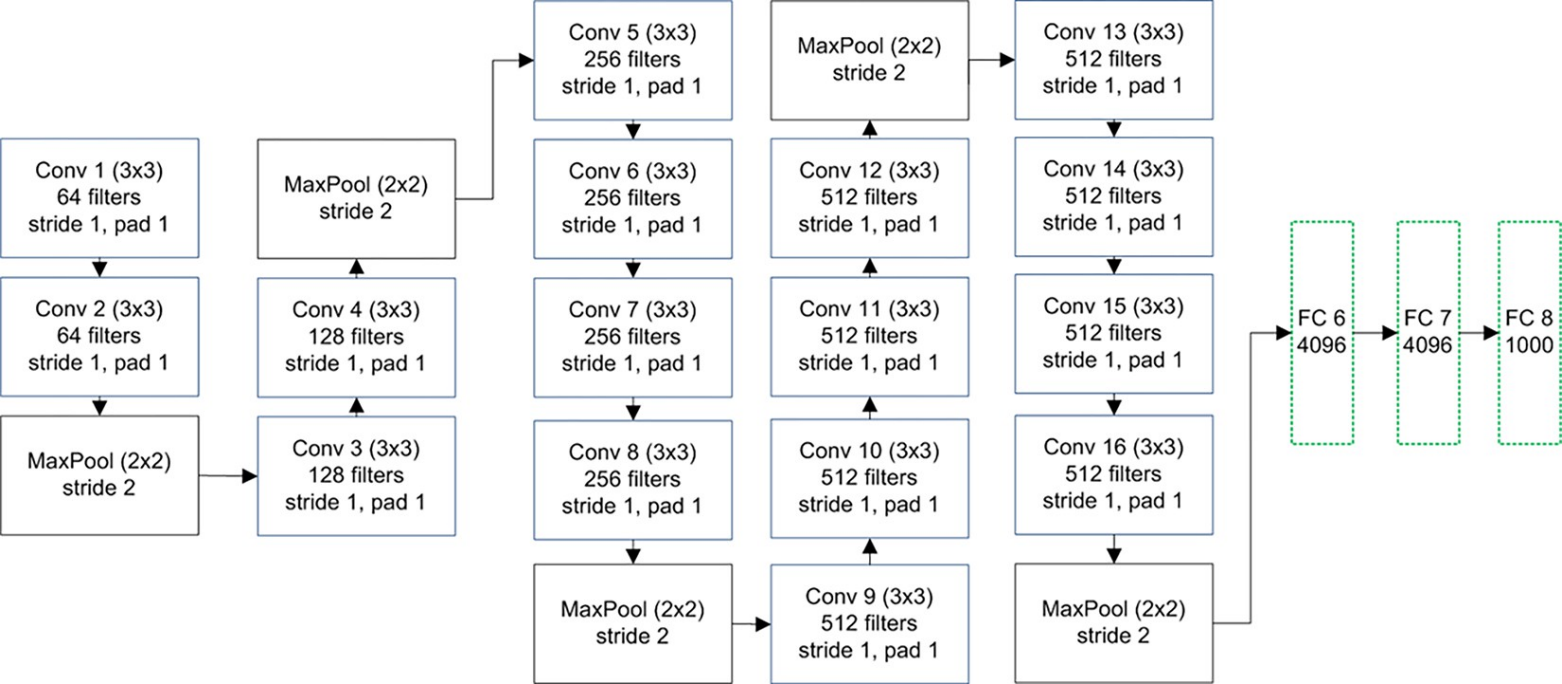
VGG-19 architecture for the proposed method.

VGG architecture is presented by the visual geometry group [51] in 2014. Its two variants are introduced i.e. 16 and 19 based on the depth of layers. Because of fewer parameters, deeper layers, uniform architecture, and small size convolution filter as compared to AlexNet, the proposed method uses the VGG-19 architecture. It comprises 16 convolution layers and 3 fully connected layers (Fig 3) and utilizes a 3×3 filter at all the convolution layers to learn as many complex features as possible and doubles its number after pooling layers to retain spatial dimensions while increasing depth. Color images of size 224×224 are first pre-processed by subtracting mean RGB values and then forwarded to convolution layers having a stride and padding of 1 pixel. The dimensions are reduced to half through 5 max-pool layers having filter of size 2 and stride 2 with no padding, occasionally between convolution layers. After convolution and pooling block, 3 fully connected layers along with dropout with a 50% probability to discard activations are utilized where the first two layers contain 4096 features and the last layer contains 1000 features. VGG uses the ReLU activation function for non-linearity and is trained by a mini-batch stochastic gradient descent algorithm.

### 3.2 Selection of optimal features through genetic algorithm

**Fig 4:**
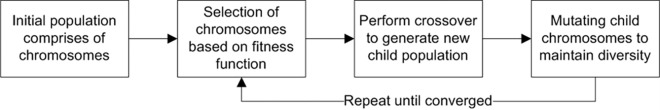
Methodology of genetic algorithm for the proposed method.

The purpose of this step is to refine the resultant feature vector by discarding irrelevant and redundant information that may affect the performance of the proposed model and end up being costly in terms of computation. Hence, a genetic algorithm (GA) [52] which is a stochastic search and optimization technique based on Darwin’s theory of natural evolution is employed in the proposed method which articulates survival of the fittest. The reason for opting for GA lies in its parallelism as it can explore an entire feature space for potential solutions/features rather than exploiting a single candidate solution and avoid being stuck in finding a locally optimal solution. The main segments of GA are i) selection (probabilistic) ii) crossover, and iii) mutation. Initially, an entire feature set extracted in the previous step is considered as a population that is encoded as real numbers represented as chromosomes. Individual components of chromosomes are called “genes”. Afterward, a probability score is calculated for each chromosome based on the fitness value calculated through the *k*-nearest neighbors (kNN) classifier [53]. In the selection process, a pair of fittest chromosomes among the entire population are selected through the roulette wheel selection method [54], where a slice of the wheel is assigned to each chromosome based on its probability value. A random pointer is attached to the wheel, which points to the chromosomes once the wheel is rotated. As the fittest chromosomes occupy a larger slice of a wheel, their chances of getting selected are higher than the ones having a minor share of a wheel. The selected pairs of chromosomes are then passed on to the crossover stage to generate a new child population. Single point crossover is applied in which genes of the parent chromosomes are swapped before and after the point which is selected randomly to get a mixture of parent’s characteristics in child chromosomes. Moreover, to get child chromosomes with distinct characteristics along with inherited ones, a mutation operation is performed. The mutation operator maintains the diversity by altering randomly selected one or more genes within child chromosomes i.e. 0 to 1 and vice versa. Fig 4 shows the overall workflow of the genetic algorithm. The above operations are repeated until the population is converged, and no distinct features are being produced further. The final feature vector after this step can be expressed as:

$$FV=\{F_{1},F_{2},\ldots ,F_{n}\}$$ (4)

### 3.3 Image classification using ELM

**Fig 5:**
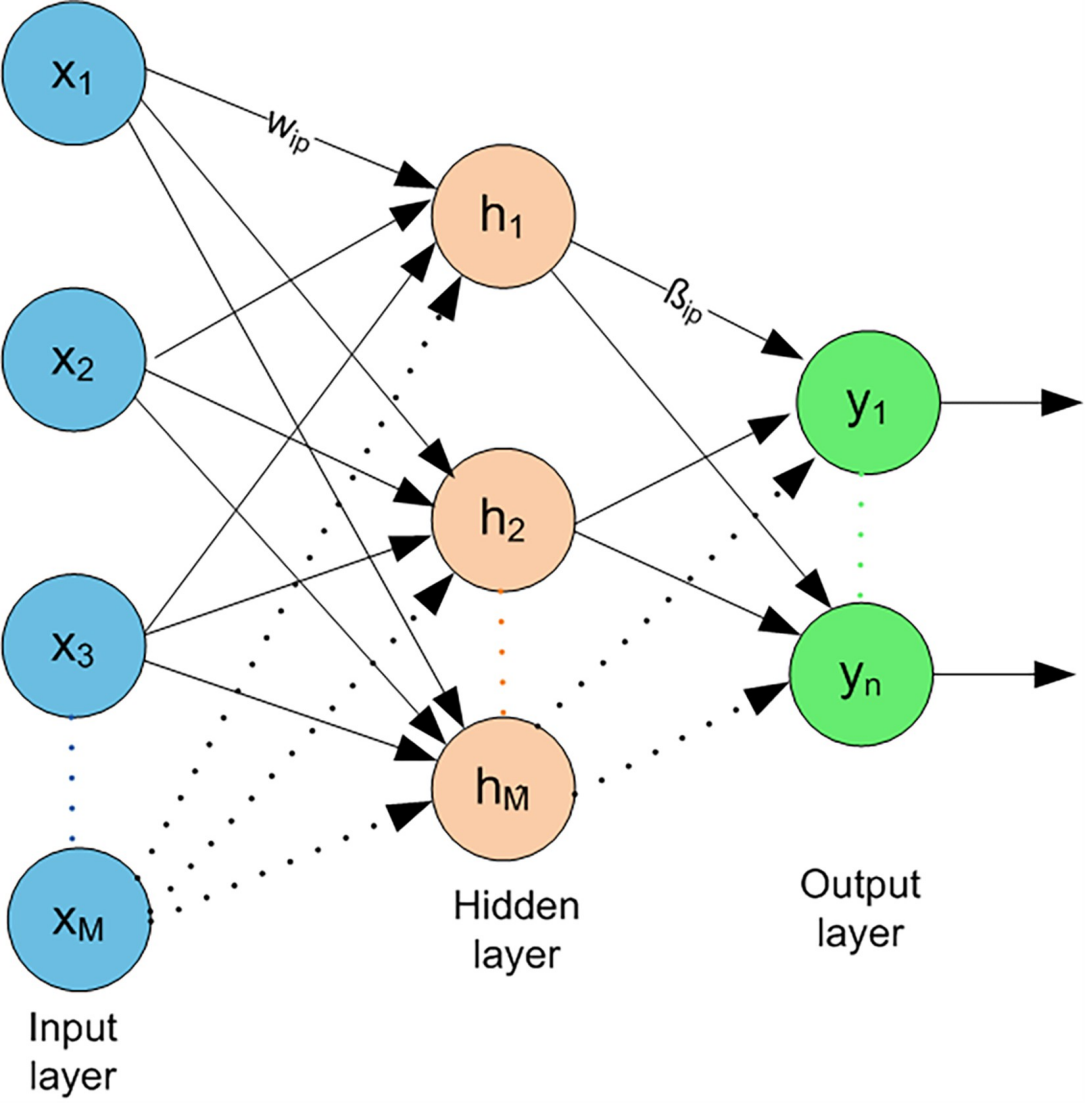
ELM schematic diagram [56].

In this step, for learning a model, the reduced feature set along with labels are passed to the extreme learning machine (ELM) classifier. The ELM is first proposed by Huang et al. [55] for single hidden layer feedforward neural networks (SLFN). Instead of fine-tuning the weights of a hidden layer using traditional gradient-based methods, the parameters of hidden nodes can be initialized randomly and need not be tuned. Hence, this makes it a linear problem whose output weights can be determined easily by applying any generalized inverse operation on the hidden layer’s output matrices. The schematic diagram of ELM classifier is shown in Fig 5. For *M* distinct samples (*x*_*i*_, *y*_*i*_)∈*R*^*d*^×*R*^*n*^, ELM classifier (one output node) having $\hat{M}$ hidden nodes and activation function *α*(*x*) can be modeled as follows:

$$f_{\hat{M}}(x)=\sum_{i=1}^{\hat{M}}\beta_{i}\alpha (x_{p})=\sum_{i=1}^{\hat{M}}\beta_{i}\alpha ({w_{i}∙x}_{p}+b_{i})=y_{p},\;p=(1,2,\cdots ,M)$$ (5)

where *β*_*i*_ = {*β*_1_,…,*β*_*n*_} is the weight vector having output weights between nodes of the output layer and *i*^*th*^ hidden node and *w*_*i*_ = {*w*_1_,…,*w*_*M*_} is the weight vector connecting input nodes with *i*^*th*^ hidden nodes. *b*_*i*_ is the thresholding value for *i*^*th*^ hidden node. The above equation can be represented in matrix form as

Hβ=Y (6)

where *H* represents the hidden layer output matrix, which is expressed as

$$\begin{array}{c} H=\left[\begin{array}{c} h(x_{1})\\ \vdots \\ h(x_{M}) \end{array}\right]={[\begin{array}{ccc} \alpha ({w_{1}∙x}_{1}+b_{1}) & \cdots & \alpha ({w_{\hat{M}}∙x}_{1}+b_{\hat{M}})\\ \vdots & \ddots & \vdots \\ \alpha ({w_{1}∙x}_{M}+b_{1}) & \cdots & \alpha ({w_{\hat{M}}∙x}_{M}+b_{\hat{M}}) \end{array}]}_{M\times \hat{M}}\\ \beta ={\left[\begin{array}{c} \beta_{1}^{T}\\ \vdots \\ \beta_{\hat{M}}^{T} \end{array}\right]}_{\hat{M}\times n}and\;Y={\left[\begin{array}{c} y_{1}^{T}\\ \vdots \\ y_{M}^{T} \end{array}\right]}_{N\times n} \end{array}$$ (7)

For better generalization, ELM aims to minimize training error ‖*Hβ*−*Y*‖^2^ and norms of output weights ‖*β*‖. The value of *β* can be evaluated as

$$\beta =H^{†}Y$$ (8)

where *H*^†^ represents the Moore–Penrose generalized inverse of output matrix *H* which can be calculated through iterative methods, singular value decomposition, or orthogonal projection methods. For a multi-class classification problem to find an optimal solution, the objective function of ELM can be formulated as.

$$\begin{array}{c} minimize\;L=\frac{1}{2}{\left\|\beta \right\|}^{2}+C\frac{1}{2}\sum_{e=1}^{M}\left\|\xi_{e}^{2}\right\|\\ subject\;to\;h(x_{e})\beta ={y_{e}}^{T}-{\xi_{e}}^{T}\;e=1,2,\ldots ,M \end{array}$$ (9)

where *ξ* represents the training error and *C* is a tunable parameter that manages the distance between the margin line and *ξ*. While training an ELM, the following dual optimization problem needs to be solved which is based on the Karush–Kuhn–Tucker (KKT) theorem.

$$L=\frac{1}{2}{\left\|\beta \right\|}^{2}+C\frac{1}{2}\sum_{e=1}^{M}\left\|\xi_{e}^{2}\right\|-\sum_{e=1}^{M}\sum_{k=1}^{n}L_{e,k}(h(x_{e})\beta_{k}-y_{e,k}+\xi_{e,k})$$ (10)

where ℒ is the Lagrange multiplier. Corresponding optimality conditions based on KKT are as follows.

$$\frac{\partial L}{\partial \beta_{k}}=0\to \beta_{k}=\sum_{e=1}^{M}L_{e,k}h{\left(x_{e}\right)}^{T}\to \beta =H^{T}L$$ (11)

$$\frac{\partial L}{\partial \xi_{e}}=0\to L_{e}=C\xi_{e}\;e=1,\ldots ,M$$ (12)

$$\frac{\partial L}{\partial L_{e}}=0\to h(x_{e})\beta -y_{e}^{T}+\xi_{e}^{T}=0\;e=1,\ldots ,M$$ (13)

where $L={\left[L_{1},L_{2},\ldots ,L_{M}\right]}^{T}$ and $L_{e}={\left[L_{e,1},L_{e,2},\ldots ,L_{e,n}\right]}^{T}$. By solving these equations, the final value of *β* becomes

$$\beta =H^{T}{\left(\frac{I}{C}+HH^{T}\right)}^{-1}Y$$ (14)

The final output of the ELM classifier can be expressed mathematically as follows:

$$f(x)=h(x)\beta =h(x){\left(\frac{I}{C}+HH^{T}\right)}^{-1}H^{T}Y$$ (15)

The class label to which the pattern **x** belongs is determined by the index of the output node with the largest output value.

### 3.4 Retrieval of the images

In this step, images are retrieved from the image database by measuring the similarity between query image *q* and dataset images *d* using Canberra distance which is mathematically defined as follows:

$$D(q,d)=\sum_{s=1}^{n}\frac{\left|q_{s}-d_{s}\right|}{\left|q_{s}|+|d_{s}\right|}$$ (16)

## 4. Evaluation parameters, results, and discussion

This section discusses in detail the chosen image datasets along with evaluation parameters that are used to assess the performance of the proposed method. A thorough discussion regarding attained results is also presented in subsequent sections.

### 4.1 Performance evaluation parameters

#### 4.1.1 Precision and recall

Precision and recall are among the frequently used performance evaluators in the CBIR framework. Precision depicts the accuracy of a system by measuring the relevancy of images against retrieved images for a certain query *q* whereas, recall depicts the robustness of the system by identifying all relevant images within a dataset.

$$P=X_{r}/X_{t}$$ (17)

$$R=X_{r}/X_{dt}$$ (18)

where *X*_*r*_ represents no. of images retrieved as relevant, *X*_*t*_ represents total retrieved images and *X*_*dt*_ represents no. of relevant images in a dataset.

#### 4.1.2 Average precision and mAP

The average of precision values against a set of queries *Q* is known as average precision which is calculated as:

$$AvgP=\frac{1}{Q}\sum_{n=1}^{Q}P(n)$$ (19)

Whereas the mean of average precision is referred to as *mAP* which is calculated as follows:

$$mAP=\frac{1}{K}\sum_{n=1}^{K}AvgP(n)$$ (20)

#### 4.1.3 F-measure

Another statistical measure that highlights the accuracy of a system and captures the properties of both precision and recall is called F-measure. Mathematically expressed as:

$$F‐measure=2*\frac{\left(P*R\right)}{P+R}$$ (21)

### 4.2 Experimental results and discussions

The proposed image retrieval framework is evaluated on 5 image datasets which are Wang-A, Wang-B, Wang 10k, OT Scene, and Caltech-256. 70% of the images are used for training and the remaining 30% are used for testing purposes from each dataset. The subsequent sections present details of each dataset along with retrieval results.

#### 4.2.1 Performance assessment on the Wang-A dataset

**Fig 6:**
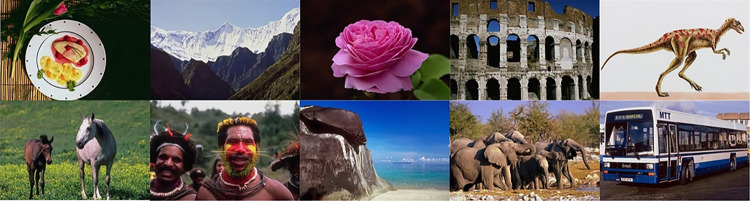
Sample images of Wang-A dataset (reprinted from [3] under a CC BY license, with permission from J. Z. WANG, original copyright [2003]).

In the CBIR domain, Wang-A [57] is one of the widely used image collections which comprises a variety of images categorized into 10 semantic classes about 100 images for each semantic class. The resolution sizes of this image collection are 256×384 or 384×256. Fig 6 shows sample images from each class.

**Fig 7:**
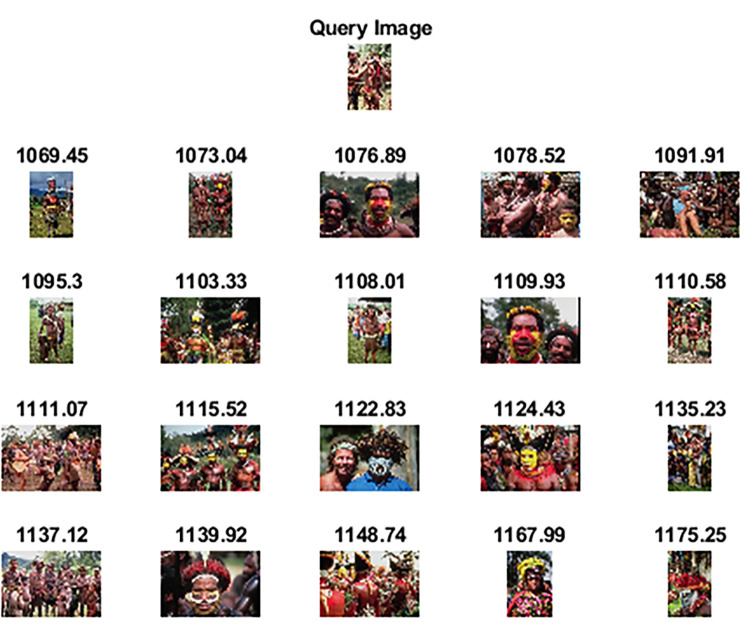
Top-20 retrieved images against query image (class: African tribes) (reprinted from [3] under a CC BY license, with permission from J. Z. WANG, original copyright [2003]).

**Fig 8:**
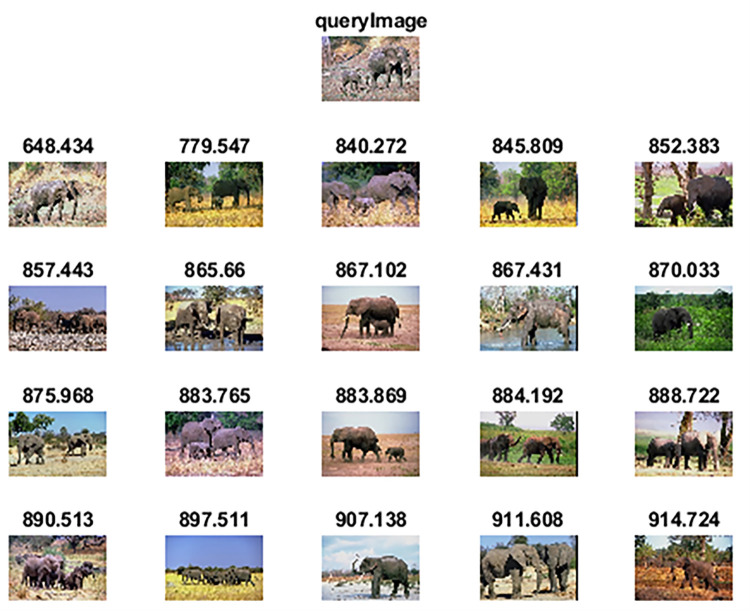
Top-20 retrieved images against query image (class: Elephants) (reprinted from [3] under a CC BY license, with permission from J. Z. WANG, original copyright [2003]).

The experimental analysis based on precision, recall, and f-measure of the proposed method along with other competitive CBIR methods is presented in Table 2. As observed in Table 2, the proposed method exhibits the best performance among all because of a) optimal deep features obtained through genetic algorithm instead of handcrafted features which require considerable human effort in the feature selection process, b) an extreme learning machine classifier which is computationally fast as compared to traditional classifiers in CBIR because of its feedforward pass approach. In many groups of Wang-A dataset, the proposed method shows promising results and has also achieved the highest precision among all competitive methods. Fig 7 shows a query image of class “African tribe” which has distinct features and the top-20 images which are retrieved as relevant against the query image by the proposed method. Fig 8 shows the top-20 retrieved images against a query image taken from class “Elephants”. The label above the retrieved images is the classification score calculated through Canberra distance. Images having lesser distance are in initial rows and are most similar in content to the query image. Against our proposed method, the precision/recall values of competitive methods on some of the classes of this dataset are better because of the complex nature of these classes. However, the overall average precision and mean recall scores highlight the better performance of our proposed method. The performance of the proposed method has also been statistically evaluated by utilizing the nonparametric Wilcoxon matched-pairs signed-rank test. Results of the nonparametric Wilcoxon matched-pairs signed-rank test are reported in Table 2. The level of significance is set at 0.05.and the results are analyzed in terms of z-value and p-value. As the p-values against all the competitor methods along with [1] are less than the level of significance, we can conclude that the proposed method shows robust performance.

**Table 2:** Performance comparison of the proposed method with state-of-the-art methods on the Wang-A dataset (values presented in bold are significant among competitive methods).

Semantic Classes		FIF-IRS [14]	VGG-16 [58]	SCNN-ELM [15]	AlexNet [59]	CM-LBP-CED [16]	Proposed Method
African Tribes	P	82.00	96.06	70.00	93.33	81.00	84.85
	R	16.40	19.21	14.00	18.66	16.20	16.97
	F	27.33	32.00	23.33	31.10	27.00	28.28
Beaches	P	60.00	84.19	66.00	90.00	66.00	87.50
	R	12.00	16.83	13.20	18.00	13.20	17.50
	F	20.00	28.00	22.00	30.00	22.00	29.16
Building	P	67.00	87.30	72.00	96.67	78.75	100
	R	13.40	17.46	14.40	19.33	15.75	20.00
	F	22.33	29.10	24.00	32.21	26.25	33.33
Buses	P	95.00	100	70.00	100	96.25	100
	R	19.00	20.00	14.00	20.00	19.25	20.00
	F	31.66	33.33	23.33	33.33	32.08	33.33
Dinosaurs	P	100	97.99	78.00	100	100	100
	R	20.00	19.59	15.60	20.00	20.00	20.00
	F	33.33	32.65	26.00	33.33	33.33	33.33
Elephants	P	95.00	91.60	96.00	100	70.75	100
	R	19.00	18.32	19.20	20.00	14.15	20.00
	F	31.66	30.53	32.00	33.33	23.58	33.33
Flowers	P	100	98.03	96.00	96.67	95.75	100
	R	20.00	19.60	19.20	19.33	19.15	20.00
	F	33.33	32.66	32.00	32.21	31.91	33.33
Horses	P	100	100	82.00	100	98.75	100
	R	20.00	20.00	16.40	20.00	19.75	20.00
	F	33.33	33.33	27.33	33.33	32.91	33.33
Mountain	P	63.00	90.70	67.00	83.83	67.75	93.33
	R	12.60	18.14	13.40	16.76	13.55	18.66
	F	21.00	30.23	22.33	27.93	22.58	31.10
Foods	P	71.00	100	100	96.83	77.25	100
	R	14.20	20.00	20.00	19.36	15.45	20.00
	F	23.66	33.33	33.33	32.26	25.75	33.33
**mAP (%) Avg. R Avg. F**	P	83.30	94.58	79.70	95.73	83.22	**96.57**
	R	16.66	18.91	15.90	19.14	16.64	**19.31**
	F	27.76	31.51	26.51	31.90	27.73	**32.18**
**Statistical analysis using non-parametric Wilcoxon matched-pairs signed-rank test**							
z-value		-2.8031	-1.9876	-2.8031	-1.9876	-2.8031	-2.8031
p-value		0.00512	0.0466	0.00512	0.0466	0.00512	0.00512

#### 4.2.2 Performance assessment on the Wang-B dataset

**Fig 9:**
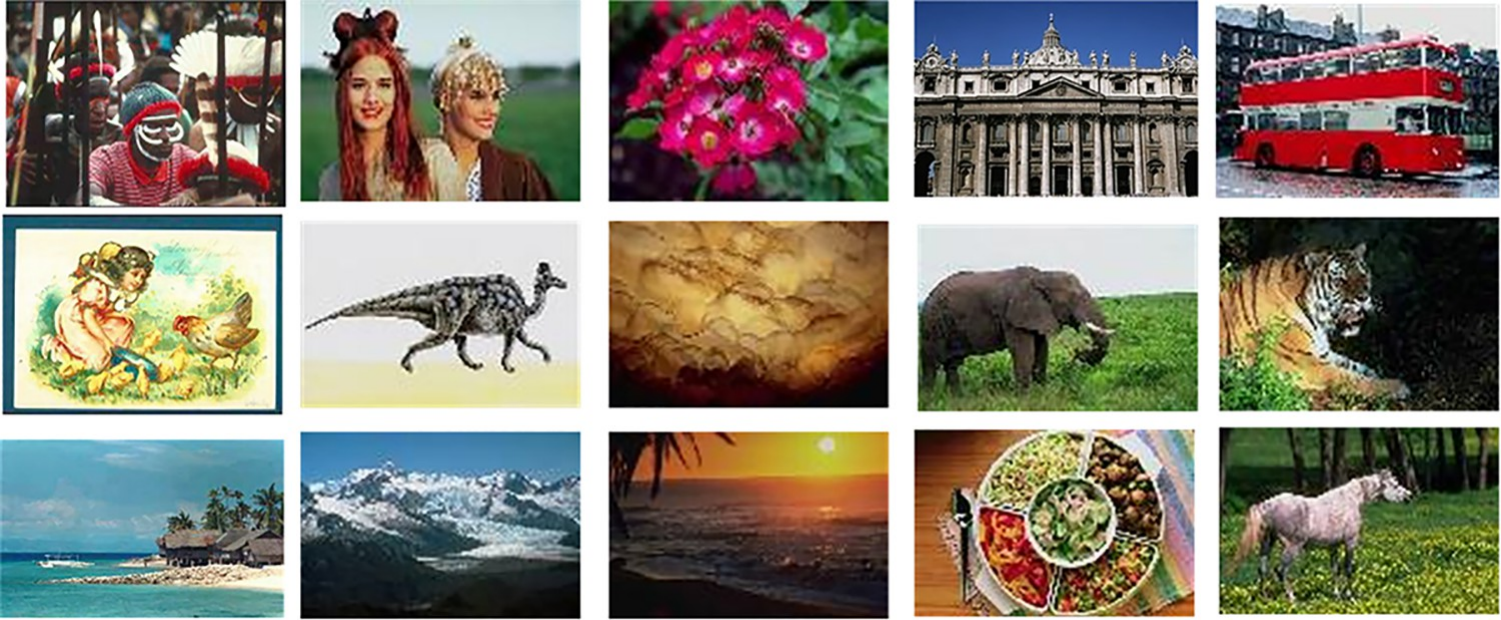
Sample images of Wang-B dataset (reprinted from [3] under a CC BY license, with permission from J. Z. WANG, original copyright [2003]).

**Fig 10:**
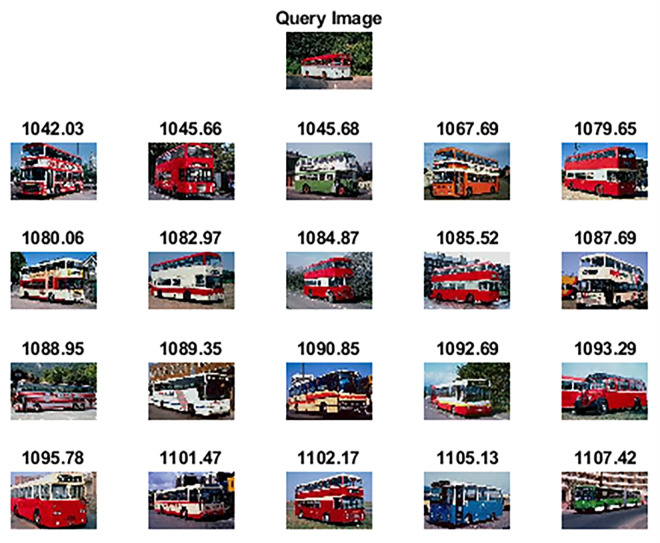
Top-20 retrieved images against query image (class: Bus) (reprinted from [3] under a CC BY license, with permission from J. Z. WANG, original copyright [2003]).

**Fig 11:**
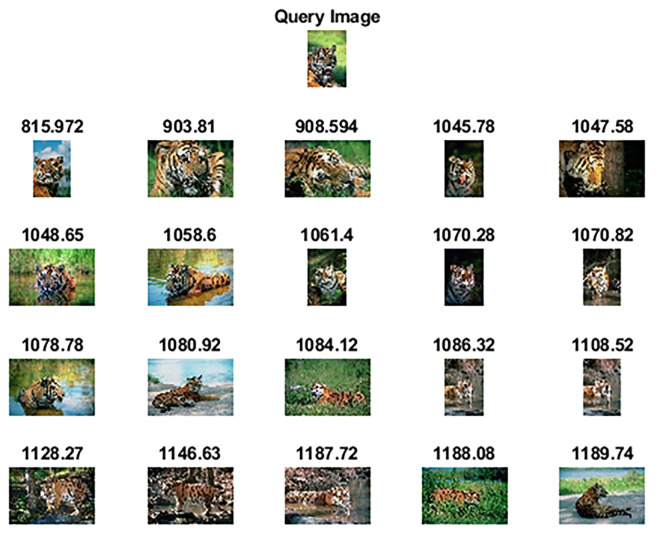
Top-20 retrieved images against query image (class: Tiger) (reprinted from [3] under a CC BY license, with permission from J. Z. WANG, original copyright [2003]).

The Wang-B [57] is another subset of the WANG dataset and comprises 15 semantic classes having 100 images each and a resolution of 256×384 pixels or 384×256 pixels. Fig 9 shows sample images of each category. Table 3 highlights the achieved results of the proposed method against other competitive methods. As shown in Table 3, the proposed method attains 91.05% precision in retrieving relevant images. The statistical analysis has also shown significant results as all the p-values are less than 0.05 when compared against competitor methods and [60]. Figs 10 and 11 show the top-20 retrieved images against query images taken from classes of “Bus” and “Tiger”. The performance of the Wang-B dataset in terms of retrieval time against a query image is 4.86 seconds as compared to the approach presented by Amsa et. al. [61] which took 32.87 seconds.

**Table 3:** Performance comparison of the proposed method with state-of-the-art methods on the Wang-B dataset.

Performance metrics	GMM-mSpatiogram [17]	SIFT-SURF [18]	LIOP-LBPV [39]	CM-DWT-CEDD [19]	Proposed Method
mAP	74.10	74.95	76.02	86.33	**91.05**
Avg. recall	13.80	14.99	15.20	17.26	**18.21**
Avg. F-measure	23.26	24.98	25.33	28.76	**30.35**
**Statistical analysis using non-parametric Wilcoxon matched-pairs signed-rank test**					
z-value	-2.8030	-2.8031	-2.8032	-2.8036	-2.8031
p-value	0.00512	0.00512	0.00514	0.00517	0.00512

#### 4.2.3 Performance assessment on the Wang 10k dataset

**Fig 12:**
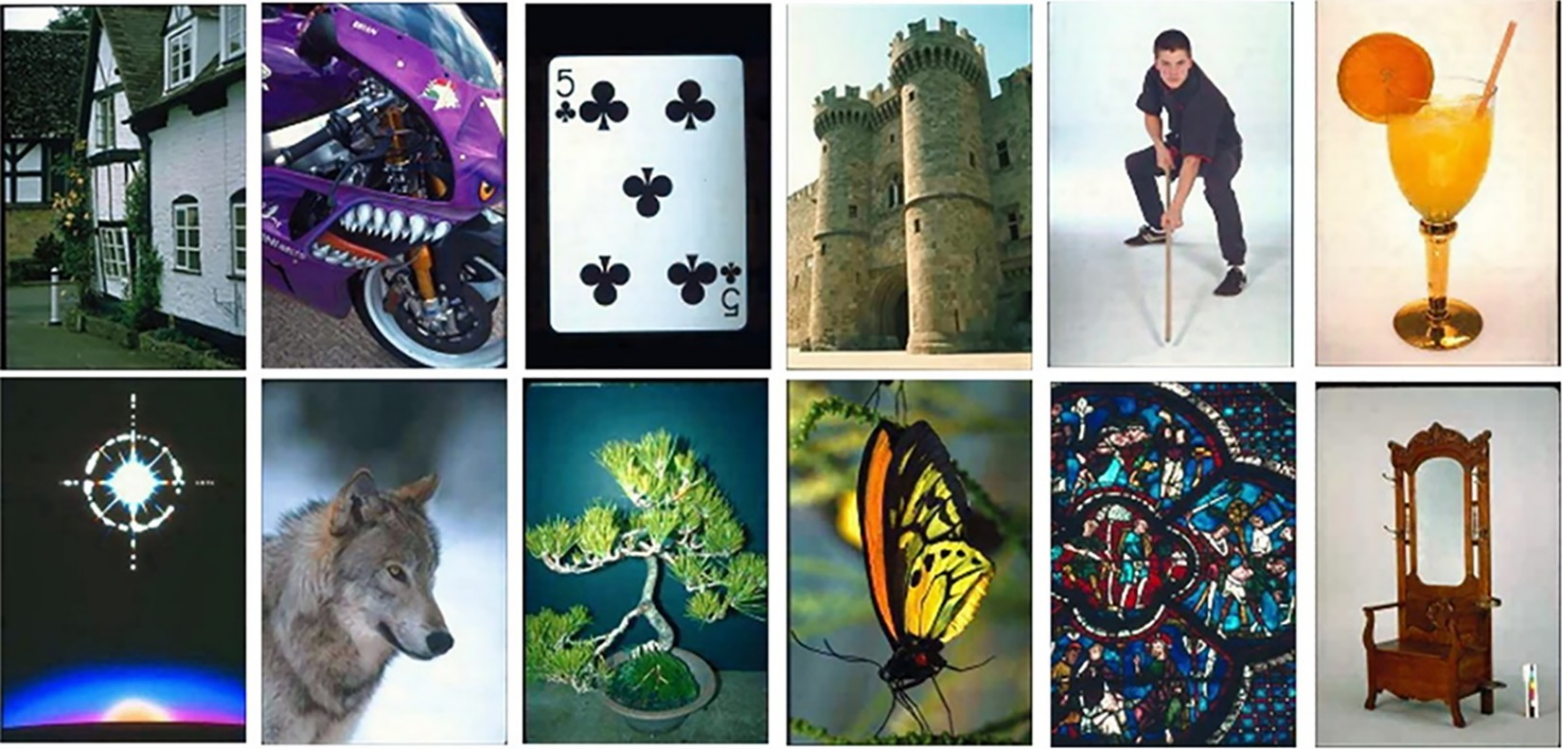
Sample images of the Wang 10k dataset (reprinted from [3] under a CC BY license, with permission from J. Z. WANG, original copyright [2003]).

**Fig 13:**
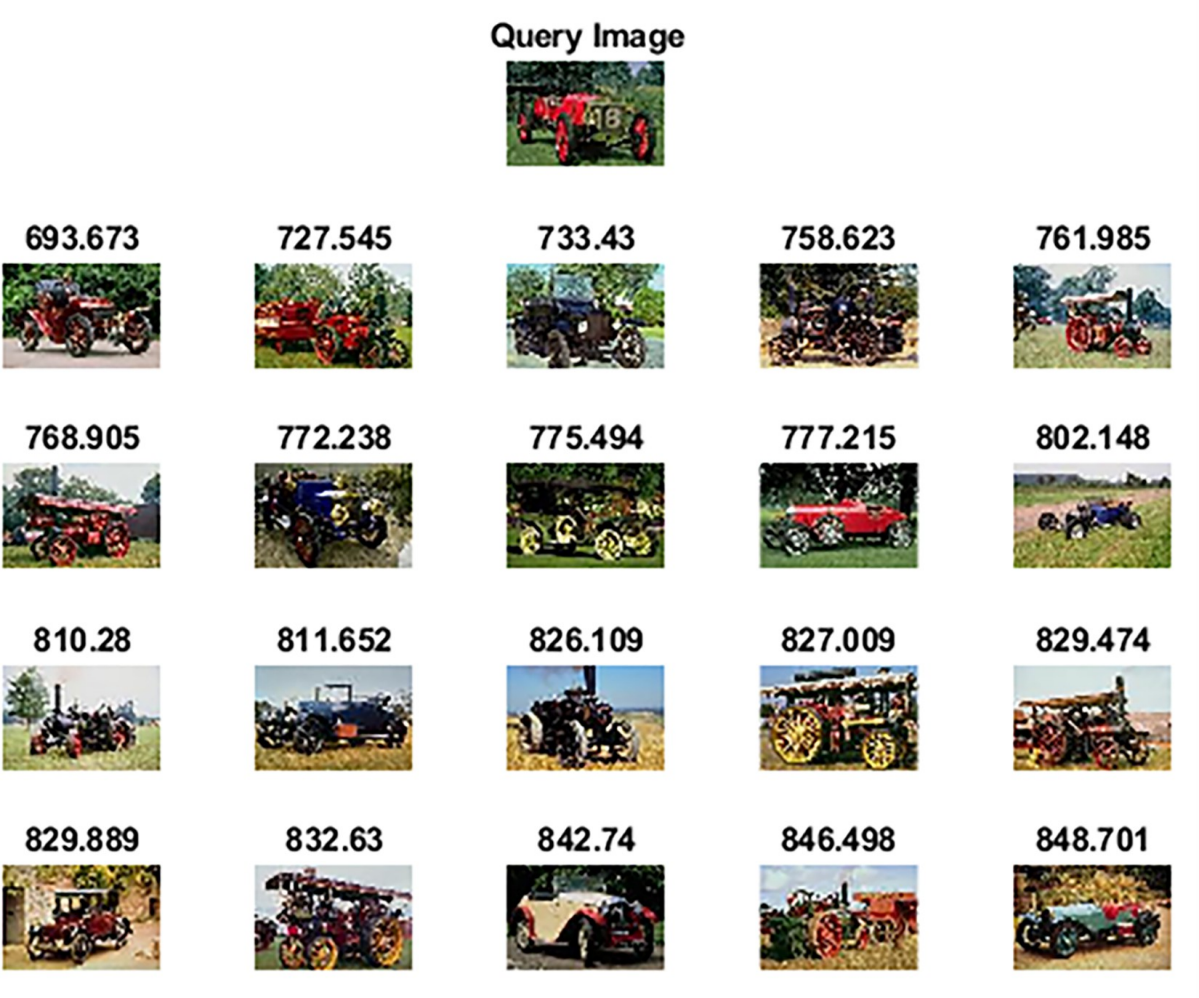
Top-20 retrieved images against a query image (class: Car) (reprinted from [3] under a CC BY license, with permission from J. Z. WANG, original copyright [2003]).

**Fig 14:**
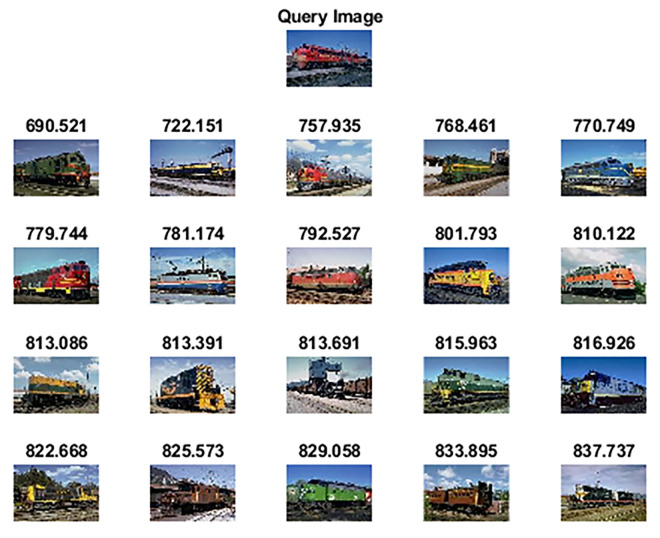
Top-20 retrieved images against a query image (class: Train) (reprinted from [3] under a CC BY license, with permission from J. Z. WANG, original copyright [2003]).

Wang 10k [62] dataset comprises 10,000 images categorized into 100 categories. Each category has 100 images of size 192×128 or 128×192 pixels. Some of the categories are ships, elephants, horses, trains, cards, butterflies, roses, mountains, sunset, musical instruments, judo-karate, etc. Fig 12 shows sample images of each category. Table 4 highlights the performance of the proposed method against other competitive methods. As shown in Table 4, the proposed method attains 78.65% precision in retrieving relevant images. Figs 13 and 14 shows the top-20 retrieved images against a query image. The p and z values of the nonparametric Wilcoxon matched-pairs signed-rank test have also shown the significant performance of our proposed method as compared to competitive methods and [63].

**Table 4:** Performance comparison of the proposed method with state-of-the-art methods on the Wang 10k dataset.

Performance metrics	GLCM-GSF-HSVCM [14]	CM-LBP-CED [16]	PUD [20]	N3G-MFR[21]	ResNet [22]	Proposed Method
mAP	56.4	59.98	58.46	65	74.60	**78.65**
Avg. recall	11.28	11.99	11.69	13	14.92	**15.73**
Avg. F-measure	18.8	19.98	19.48	21.66	24.86	**26.21**
**Statistical analysis using non-parametric Wilcoxon matched-pairs signed-rank test**						
z-value	-2.8031	-2.8033	-2.8032	-2.8035	-2.8037	-2.8031
p-value	0.00512	0.00512	0.00512	0.00513	0.00515	0.00512

#### 4.2.4 Performance assessment on the OT scene dataset

**Fig 15:**
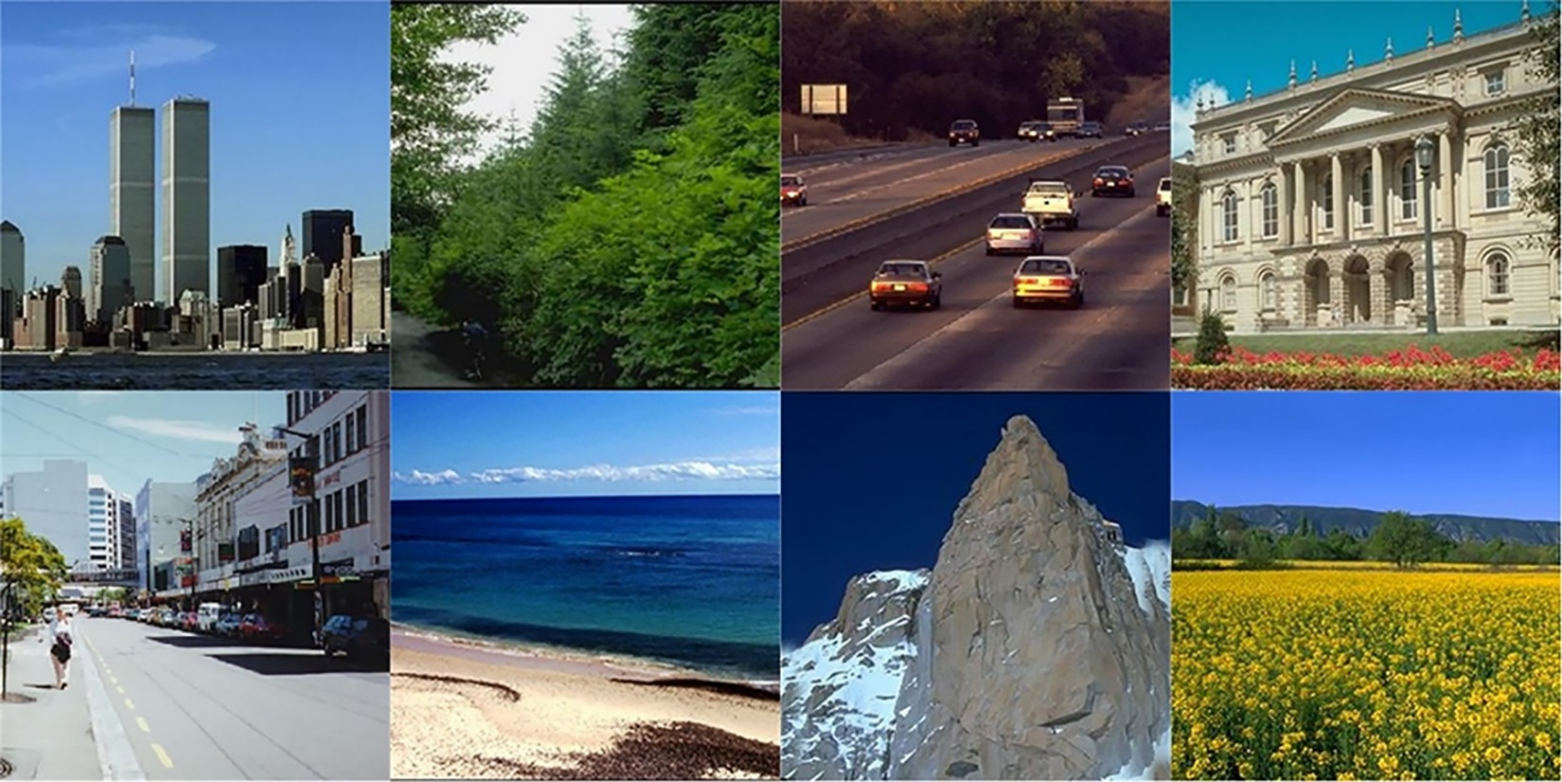
Sample images of the OT Scene dataset [64].

The OT scene [64] dataset consists of 2688 images which are divided into 8 different categories. Each category has varying no. of images of size 256×256. Categories include coast, beach, forest, open country, mountain, highway, street, city center, and tall building. Fig 15 shows some sample images from each class of the OT scene dataset.

**Fig 16:**
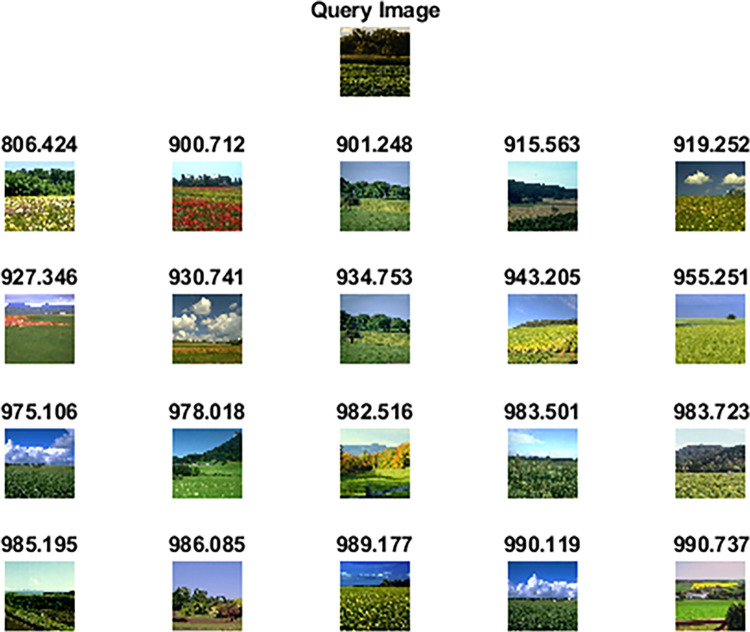
Top-20 retrieved images according to the query image of the OT Scene dataset (class: open country).

**Fig 17:**
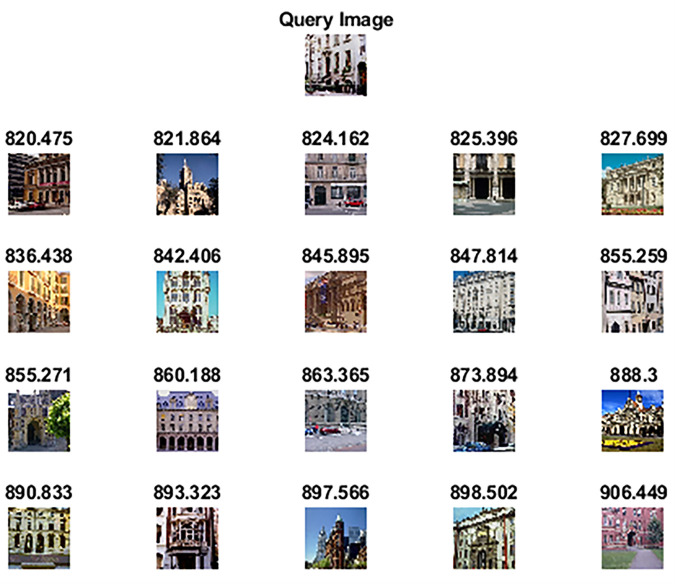
Top-20 retrieved images according to the query image of the OT Scene dataset (class: inside a city).

Table 5 shows the performance of our proposed method on the OT scene dataset against competitive methods. Figs 16 and 17 shows the top 20 images retrieved against query image which belongs to the class “open country” and “inside city”. The statistical analysis has also shown significant results as all the p-values are less than 0.05 when compared against competitor methods and [65].

**Table 5:** Performance comparison of the proposed method with state-of-the-art methods on the OT Scene dataset.

Performance metrics	CDH-ART [23]	B-T-Morph [24]	SIFT-SURF [18]	HWVP[25]	ISA-SPM [26]	FC-GPHOG [27]	Proposed Method
mAP	51.04	60.7	69.75	77.2	86.29	89.6	**90.36**
Avg. recall	10.20	12.14	13.95	15.44	17.25	17.92	**18.07**
Avg. F-measure	17	20.23	23.25	25.73	28.75	29.86	**30.11**
**Statistical analysis using non-parametric Wilcoxon matched-pairs signed-rank test**							
z-value	-2.8031	-2.8031	-2.8033	-2.8033	-2.8035	-2.8036	-2.8033
p-value	0.00512	0.00512	0.00513	0.00513	0.00514	0.00514	0.00513

#### 4.2.5 Performance assessment on the Caltech 256 dataset

**Fig 18:**
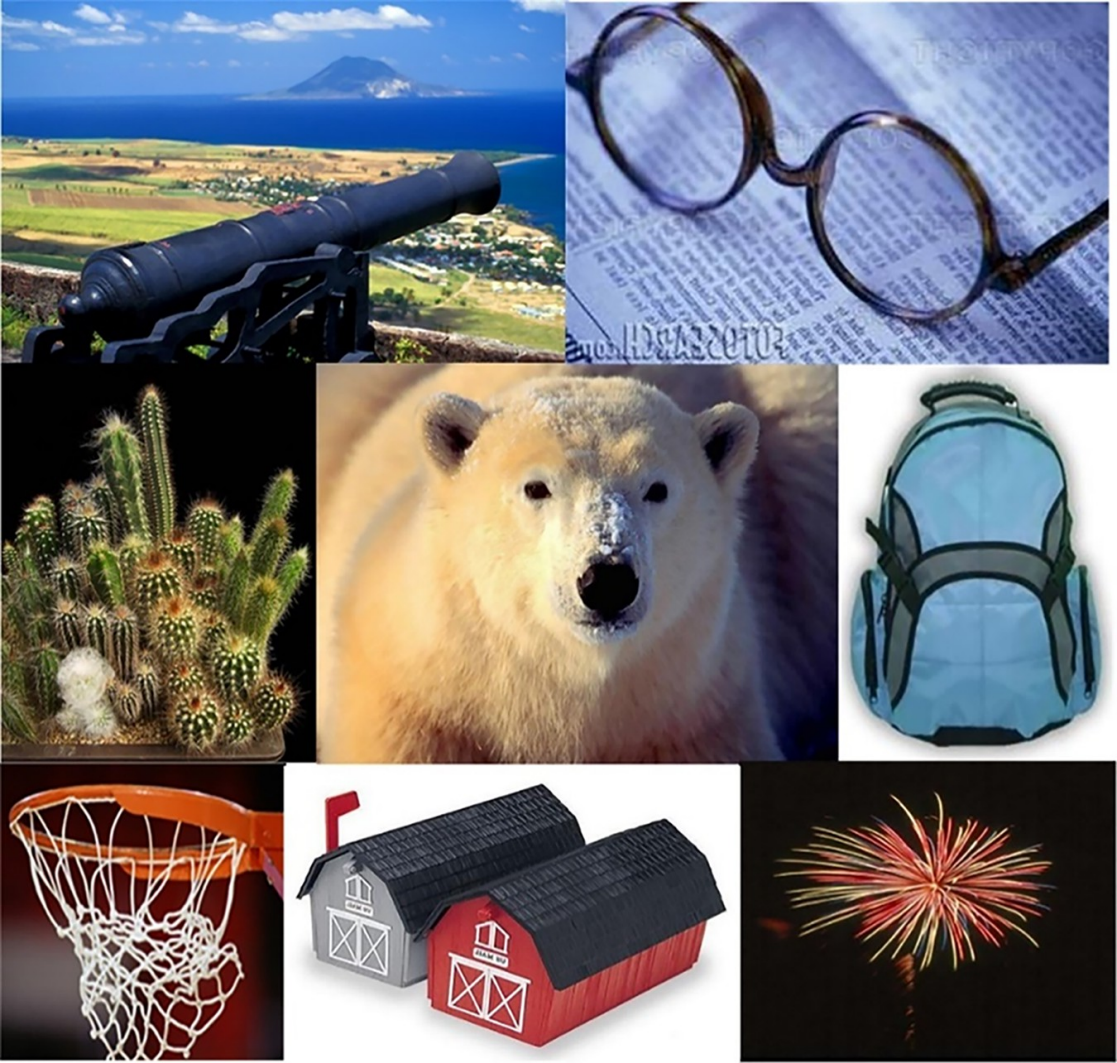
Sample images of the Caltech-256 dataset [66].

The Caltech 256 [66] dataset has a total of 30,607 images categorized into 257 object categories. Each category has at least 80 images having varied resolutions. It is a challenging dataset as compared to its predecessor Caltech-101 as more variation in object size, pose, and location is considered. Some of the sample images are shown in Fig 18.

**Fig 19:**
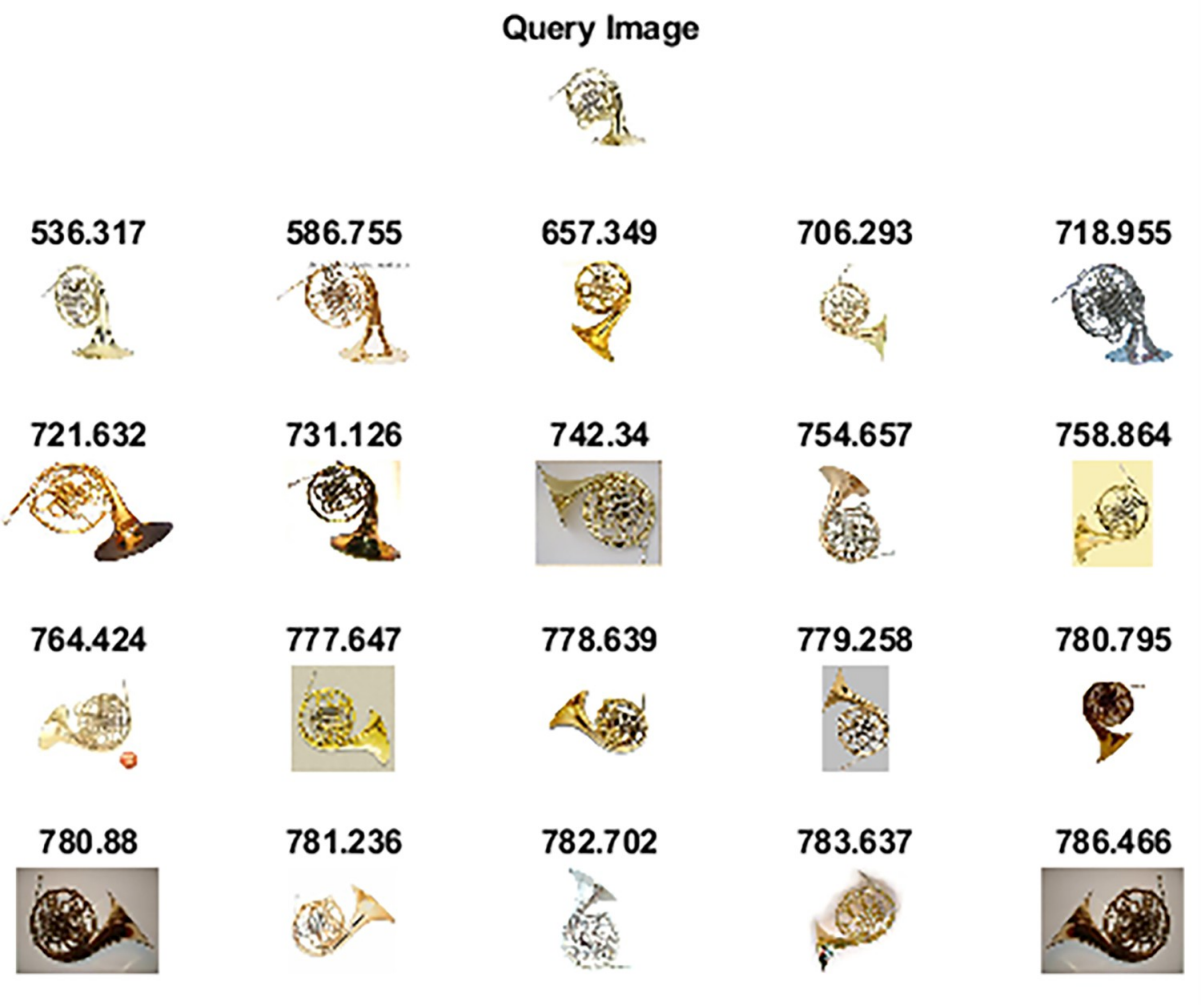
Top-20 retrieved images according to the query image of the Caltech-256 dataset (class: French Horn).

**Fig 20:**
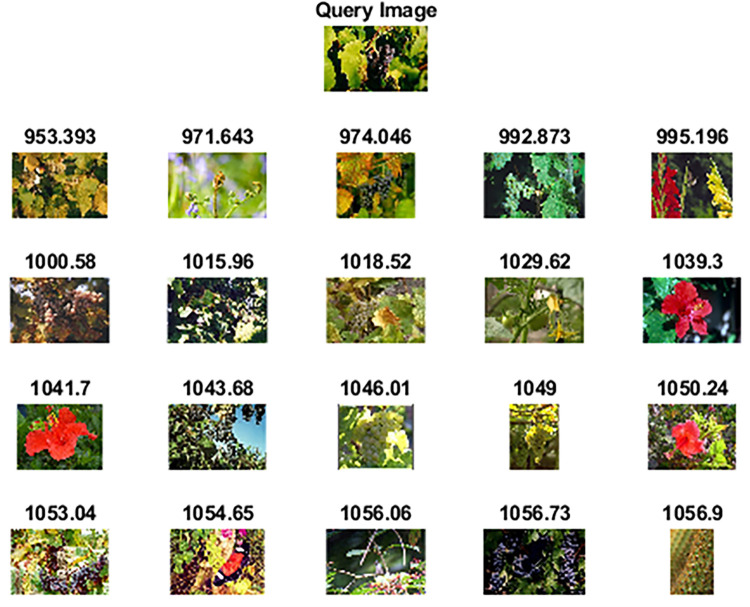
Top-20 retrieved images according to the query image of the Caltech-256 dataset (class: Grapes).

Table 6 shows a better performance of the proposed method against competitive methods as it achieves 80.95% precision as compared to other methods. Figs 19 and 20 shows the top-20 retrieved relevant images closest to the query image in terms of content. The p and z values of the nonparametric Wilcoxon matched-pairs signed-rank test have also shown the significant performance of our proposed method as compared to competitive methods as well as against [68].

**Table 6:** Performance comparison of the proposed method with state-of-the-art methods on the Caltech-256 dataset.

Performance metrics	FC-GPHOG [27]	ACEnet [67]	Balanced tree structures [28]	ResNet-HAM [29]	Proposed Method
mAP	33	36.99	38.56	74.7	**80.95**
Avg. recall	6.6	7.39	7.71	14.94	**16.19**
Avg. F-measure	11	12.31	12.85	24.9	**26.98**
**Statistical analysis using non-parametric Wilcoxon matched-pairs signed-rank test**					
z-value	-2.8025	-2.8026	-2.8027	-2.8034	-2.8027
p-value	0.00510	0.00510	0.00510	0.00513	0.00510

#### 4.2.6 Discussions of experimental results

**Fig 21:**
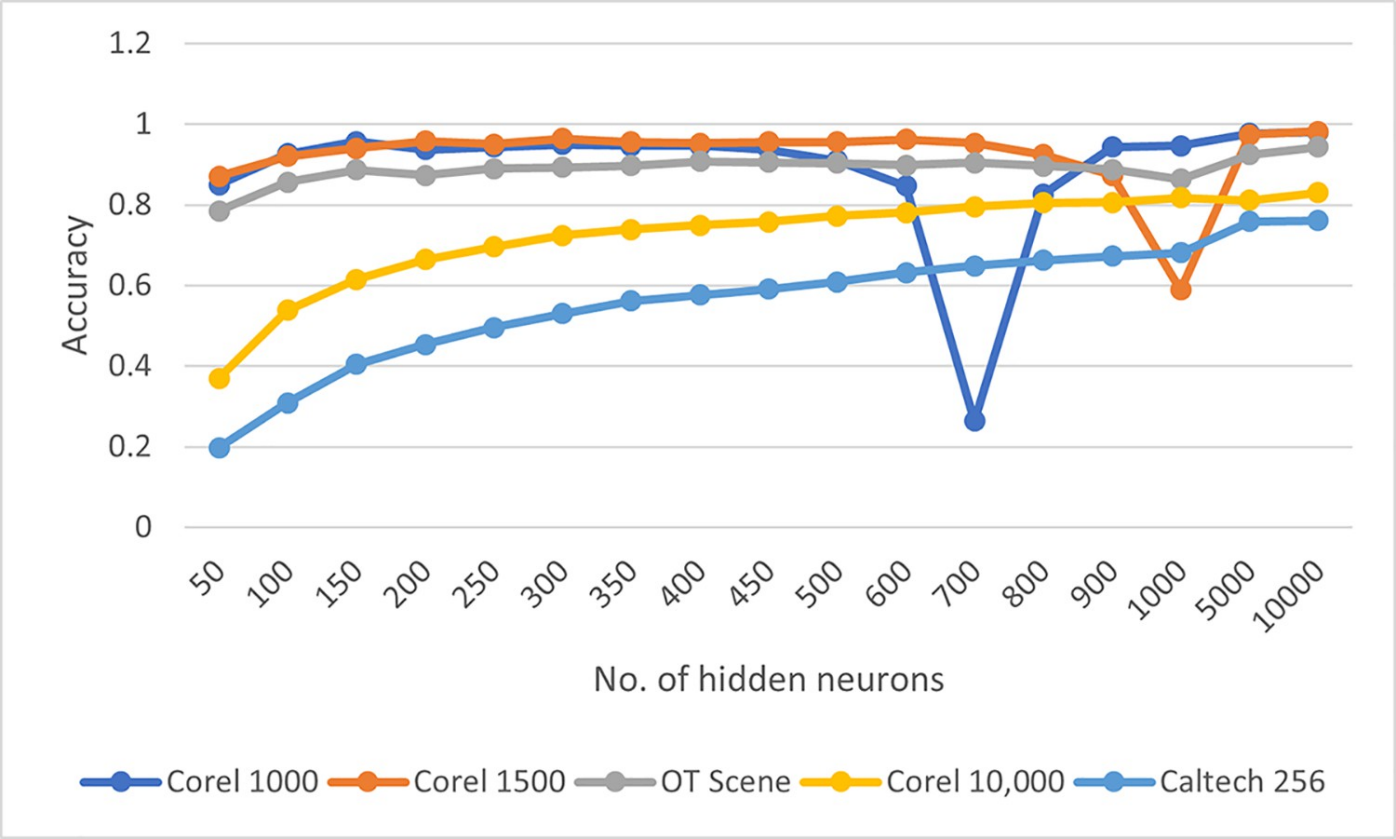
Effect of no. of hidden neurons on the accuracy of the proposed method.

**Fig 22:**
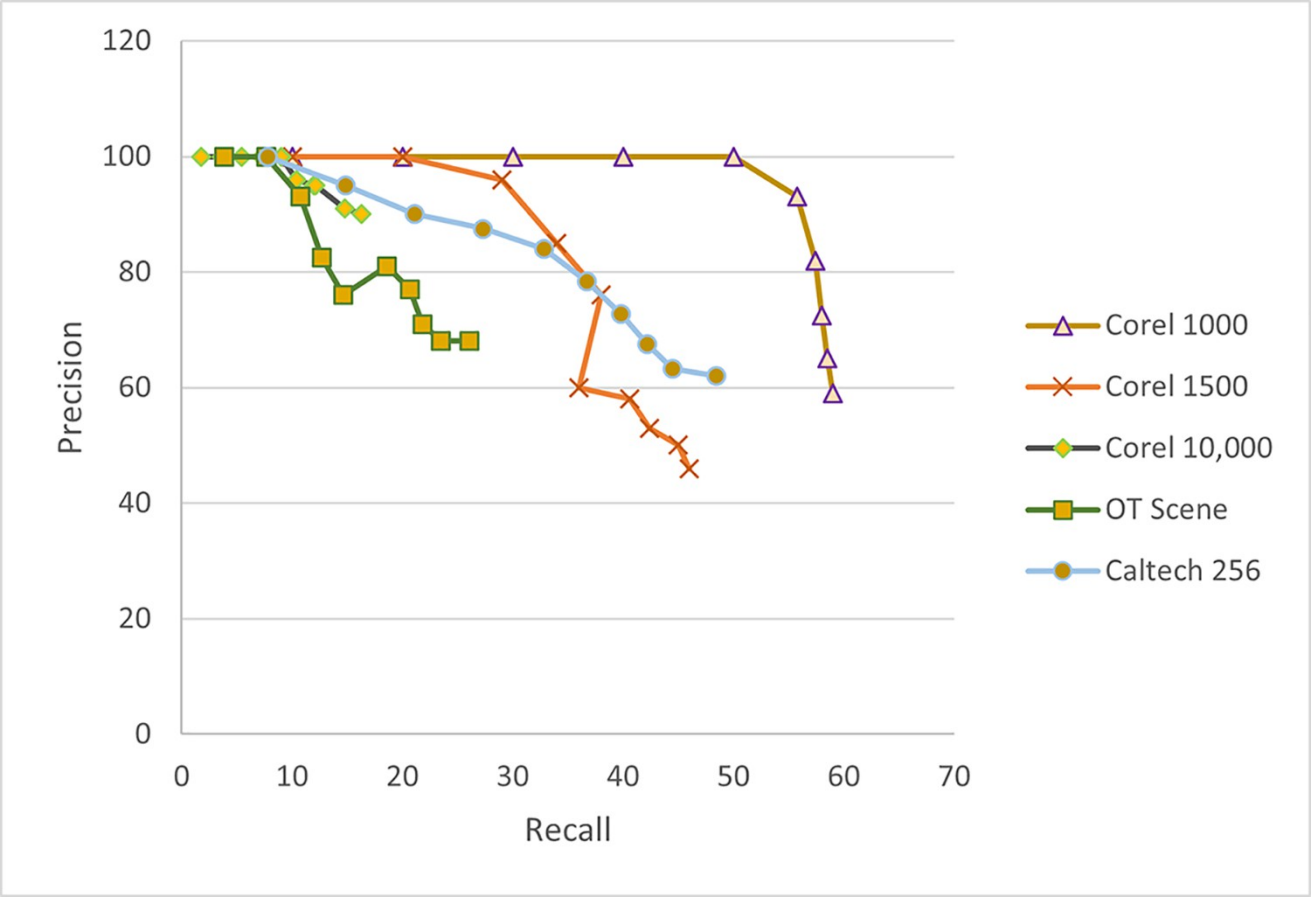
Performance analysis in terms of the precision-recall curve of the proposed method.

The reason for opting for an ELM classifier is its random independent feature transformation and quadratic loss function which guarantees the convergence of training to a global optimum solution [69]. As compared to traditional classifiers, it has fewer optimization constraints and better generalization capabilities [70]. One of the parameters to adjust in the ELM classifier is the no. of hidden neurons which can influence the retrieval accuracy of the proposed system. The reported retrieval accuracy is achieved when no. of hidden neurons is in the range of 200–300. The retrieval accuracy keeps on fluctuating between this range but gradually starts to increase when no. of neurons is set to 1000 or more. Fig 21 represents the accuracy vs no. of hidden neurons curve over selected datasets. Even though better performance is observed while increasing the no. of neurons but it resulted in increased computational time. As observed in Fig 21 accuracies of Wang-A, Wang-B, and OT datasets tend to decrease at points where no. of hidden neurons is equivalent to no. of training images. In Fig 22 it can be seen that while increasing the no. of images retrieved precision remains the same in most of the chosen datasets whereas an increase in a recall is observed when more images are retrieved, highlighting the effective performance of our proposed method.

The limitations of handcrafted approaches mentioned in sections 1–2 like limited image expressing capabilities, expensive design, etc. are addressed by utilizing a VGG-19 architecture that can learn features in an automated form. As the feature vector, we get from the FC-7 layer of the network is of a higher dimension. There needs to be a dimension reduction strategy that can not only selects the important features but also be computationally efficient while classifying the images. To address this, the incorporation of a genetic algorithm in the proposed approach not only selects the optimal features but also reduces the feature vector size. This resultant feature vector is approximately half in dimension as compared to the original feature vector. For classification, ELM being a single hidden layer feedforward neural network works better in terms of precision, recall, f-measure, and retrieval time as compared to hand-crafted methods of CBIR.

#### 4.2.7 Required resources and comparative analysis of computational cost

The hardware and software resources upon which the performance of the proposed method is assessed are as follows: a PC having Intel Core i7-7700 3.60 GHz processor, RAM 8GB, Microsoft Windows 10 (64-bit), and MATLAB 2019b (64-bit). The competitor approaches of the proposed method utilize integrated local features like color, shape, texture, etc., and global features having varying sizes of feature vectors along with clustering and classification which results in increased computations. The feature extraction and later feature fusion are not only dependent on the researcher’s knowledge but are computationally expensive as compared to CNN architectures. In the proposed method, a convolution neural network i.e., VGG-19 architecture extracts features in an automated way by convolving an image with a fixed kernel of size 3. This eases the feature engineering task as well as it is invariant to scale, rotation, and translation and computationally less expensive as compared with competitive image retrieval methods. The performance comparison in terms of the computational cost (retrieval time) of the proposed method and its competitive methods for a Wang-A dataset is presented in Tables 7 and 8 represents the retrieval time of the proposed method on the Caltech-256 dataset.

**Table 7:** Computational time (in seconds) of the proposed method and its comparative analysis with competitive methods of CBIR on the Wang-A dataset.

CM-LBP-CED [16]	FIF-IRS [14]	Spatial color-Shape [31]	DNN-SAR [35]	SURF-HOG [32]	CHLDP-DSIFT [33]	MDGHM-SURF-ORB [34]	Proposed Method
1.1087	1.46	1.34	1.26	0.7845	0.7837	0.5124	0.47

**Table 8:** Computational time (in seconds) of the proposed method and its comparative analysis with competitive methods of CBIR on the Caltech-256 dataset.

No. of images retrieved	Proposed method	DNN-SAR [35]	Spatial color-Shape [31]
10	0.28	0.93	1.06
15	0.76	1.0	1.11
20	0.9	1.07	1.19
25	0.94	1.11	1.25
30	1.01	1.16	1.26

## 5. Conclusion and future work

The most important factors for an image retrieval system to be termed efficient and accurate are its retrieval accuracy and utilization of computational resources. Reduction in feature vector dimensionality or extracting the appropriate features can influence both factors. So, the proposed method first extracts the features through VGG-19 architecture which resulted in a 4096-dimensional vector. All of these extracted features may not be useful and can consume more resources and time during execution. Hence irrelevant, and redundant features are discarded by utilizing a genetic algorithm. The proposed method used an ELM classifier because it’s computationally fast and easily trained. Classification results over 5 datasets clearly show that the proposed method has the highest precision and recall rates among other competitive CBIR methods. In the future, we’ll explore other deep architectures and different versions of the ELM classifier to enhance the CBIR process.
